# Health anxiety in frequently and rarely ill younger adolescents

**DOI:** 10.1192/j.eurpsy.2022.1090

**Published:** 2022-09-01

**Authors:** I. Shishkova, E. Pervichko

**Affiliations:** 1 Ryazan State Medical University named after I.P. Pavlov, Faculty Of Clinical Psychology, Ryazan, Russian Federation; 2 Lomonosov Moscow State University, Faculty Of Psychology, Moscow, Russian Federation; 3 Pirogov Russian National Research Medical University, Clinical Psychology Department, Moscow, Russian Federation

**Keywords:** health anxiety, frequently ill adolescents, subjective pattern of health

## Abstract

**Introduction:**

It is known that a high level of health anxiety is traditionally recognized as obligatory for hypochondria, which is characterized by a clear and pronounced belief of the subject in the presence of a disease or the danger of its development (A psychiatric glossary, 1975). Such patients are usually characterized by high concern about their health, but this anxiety can be represented by varying degrees of severity. At the same time, it is important to talk not only about pathological anxiety (hypochondria), but also about conditions associated with normal human anxiety about their health, also in children and adolescents.

**Objectives:**

To study health anxiety in younger adolescents.

**Methods:**

The sample: 101 respondents (44 rarely ill younger adolescents (mean age 10.6±0.1), 57 frequently ill younger adolescents (mean age 10.5±0.43)). We used: “Short Health Anxiety Inventory” (SHAI; Salkovskis et al., 2002), Questionnaire “Index of attitude toward health” (Deryabo, Yasvin, 1999), CPQ (Porter, Cattell, 1985).

**Results:**

The results of multiple regression analysis for a sample of younger adolescents showed that the scale of actions to preserve and promote health and factor I (sensitivity) make up the level of severity of the general scale of health anxiety in rarely ill younger teenagers (-0.476, p=0.045; 0.628, p=0.039). Health anxiety is determined by factor O (anxiety) in frequently ill younger teenagers (0.316, p=0.029).

**Conclusions:**

Health anxiety can be viewed as a non-pathological construct associated with personality traits and behavior and has structural differences depending on the diseases’ frequency. Research is supported by the Russian Science Foundation, project No. 21-18-00624.

**Disclosure:**

Research is supported by the Russian Science Foundation, project No. 21-18-00624.

